# Altering the Microstructure of Conjugated Polymers in Solution via Microwave Irradiation

**DOI:** 10.1021/acs.macromol.5c00942

**Published:** 2025-07-01

**Authors:** Chia-Chun Lin, Suhendro Purbo Prakoso, Livy Laysandra, Ming-Hao Chang, Hai-I Jo, Yen-Ting Li, Kai-Lin Chen, Chun-Yu Chen, Lewis M. Cowen, Eisuke Fujiwara, Audithya Nyayachavadi, Simon Rondeau-Gagné, Bob C. Schroeder, Shinji Ando, Wei-Tsung Chuang, Jhih-Min Lin, Yu-Cheng Chiu

**Affiliations:** † Department of Chemical Engineering, 34878 National Taiwan University of Science and Technology, No.43, Sec. 4, Keelung Rd., Daan District, Taipei 10607, Taiwan; ‡ Advanced Institute for Materials Research, 13101 Tohoku University, 2 Chome-1-1 Katahira, Aoba Ward, Sendai, Miyagi 980-8577, Japan; § 57815 National Synchrotron Radiation Research Center, 101 Hsin-Ann Road, Hsinchu Science Park, Hsinchu 30076, Taiwan; ∥ Department of Chemistry, 4919 University College London, 20 Gordon Street, London WC1H 0AJ, United Kingdom; ⊥ Department of Chemical Science and Engineering, 13290 Institute of Science Tokyo, Ookayama 2-12-1-E4-5, Meguro-ku, Tokyo 152-8552, Japan; # Department of Chemistry and Biochemistry, 8637 University of Windsor, Windsor, Ontario N9B 3P4, Canada; ∇ Sustainable Electrochemical Energy Development Center, National Taiwan University of Science and Technology, Taipei 10607, Taiwan; ○ Advanced Research Center for Green Materials Science and Technology, National Taiwan University, Taipei 10617, Taiwan

## Abstract

Solution-processed conjugated polymers can exhibit high charge carrier mobilities depending on the microstructure of the resultant films. Controlling the solution-state aggregation, however, remains challenging for mass production. Through the application of microwave (MW) heating, we demonstrate that poly­[*N*,*N*′-bis­(2-octyldodecyl)­naphthalene-1,4,5,8-bis­(dicarboximide)-2,6-diyl]-*alt*-5,5′(2,2′bithiophene) *P*(NDI2OD-T2) solution in chlorobenzene can impart a dramatic improvement in the microstructure of the thin film, rendering transistor devices with highest saturation field-effect mobility values of 0.45 cm^2^ V^–1^ s^–1^. Here, by employing *in situ* optical spectroscopies and small-angle X-ray scattering (SAXS) techniques, we elaborate the controllable swelling-collapsing dynamics of a polymer solution into ordered aggregates under MW heating. The *in situ* optical measurements, including absorption and photoluminescence (PL), reveal distinguished features of the intra/intermolecular ordering of polymer chains, while the *in situ* SAXS unveils the effectiveness of MW heating in reordering the swollen coils. As a result, MW heating offers rapid operation, high-energy efficiency, and scalable fabrication, and it is a versatile technique enhancing charge transport of the final thin films, regardless of their casting processes and conjugated polymer structures. This finding may shed insight into the mechanism of reordering aggregation on conjugated polymer solutions under MW irradiation for further research and development in related fields.

## Graphical abstract

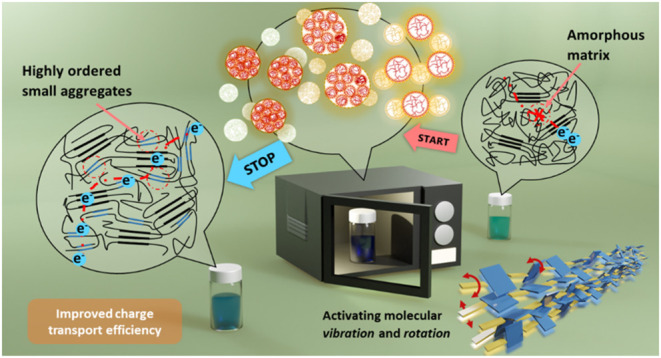

## Introduction

Conjugated polymers have garnered interest in a variety of applications, including organic field-effect transistors (OFETs), organic light-emitting diodes (OLEDs), and photovoltaic cells (PVCs). This is because conjugated polymers are cost-effective, mechanically flexible, and industrial-scale compatible, allowing for relatively low-temperature processing.^1^ Solution processability is another benefit of conjugated polymers, but relatively low solubility, compared to small molecule organic semiconductors, can hinder practical applications and investigations.^2,3^ The at times limited solubility originates from the strong intermolecular π–π interactions between the rigid and conjugated backbones of semiconducting polymers. To enhance the polymer solubility, commonly employed strategies include the introduction of solubilizing side groups,^4,5^ random polymerization,^6,7^ and structural asymmetry.^8,9^ However, the molecular structure itself influences not only solubility but also optical properties, molecular packing, and energy levels. Thus, resolving the solubility limit alongside the desired physical and chemical characteristics can be challenging. Given the numerous synthetic steps typically required to modify the chemical structure of conjugated polymers, developing an efficient method to enhance their solubility without the necessity of structural changes is highly appealing.

Moreover, in solid state, conjugated polymers often adopt complex semicrystalline microstructures with crystalline domains embedded in an otherwise amorphous matrix.^10^ Both morphological and molecular packing structure impact the charge transporting behavior, which determines the carrier mobility and index parameters for the transistor device. Within the crystalline regions, the enhanced overlap of π-orbitals yields larger charge-transfer integrals and hopping rates between the intra/interchains of the conjugated polymers. Consequently, the poorer π-orbital overlap in the amorphous region typically generates the charge scattering and trapping sites that inhibit effective charge migration. Therefore, semiconducting polymers with intrinsic conformational freedom often suffer from relatively low charge carrier transport, stemming from a low degree of π-stacking between polymer chains in the solid state. However, recent polymer designs with ever more complex monomers seem to be opposing old concepts in attaining high charge carrier mobility, such as those involving regioregularity-control, texture, or crystallinity.^11–13^ On top of this, conjugated polymers with highly disordered or fully amorphous microstructures exhibited on par or even higher carrier mobility than those of semicrystalline polymers.^11,14^ Salleo et al. reported that high carrier mobility can be achieved by virtue of the interconnected aggregates, even if on smaller scales and disordered.^15^ From their findings, rather than pursuing polymers with higher crystallinity, a polymer with high tolerance to an inevitably large amount of disorder in the aggregate state could allow for more efficient intra- and intermolecular charge transport at the segmental level of the interconnected aggregates. However, highly semicrystalline conjugated polymers have a higher tendency to cause deep traps in their π-stacking direction, which can engender low charge transport efficiency. Thus, control over the ordered aggregates in conjugated polymers is essential in ensuring an efficient intermolecular charge transfer.^15,16^

Another strategy to impart an ordered microstructure is post processing, for instance, through thermal annealing^17–19^ or solvent vapor annealing.^20^ These post-treatments utilize heat or solvent to promote polymer chain movement and subsequently diminish morphological defects. To eliminate the need for additional post processing, several methods, including dissolving the conjugated polymer in marginal solvents and sequential ultraviolet (UV) irradiation after ultrasonication,^21,22^ have been developed to induce well-ordered aggregates in solution prior to film deposition. Having said that, those external stimuli are sometimes inapplicable to a broad range of conjugated polymers for inducing direct ordering in polymer aggregates. Herein, we propose the utilization of microwave (MW) irradiation to facilitate the solvation of a conjugated polymer while simultaneously promoting the formation of well-ordered polymer aggregates in solution prior to film deposition (Figure 1). Our process involves heating the polymer solution via MW irradiation, followed by subsequent natural cooling to yield the polymer solution for film deposition. Microwave, electromagnetic waves with wavelengths ranging between 10^–3^ and 1 m are associated with rotational energy levels in molecules.^23,24^ The polarizability of molecules containing electric and/or magnetic dipoles enables efficient MW absorption. In return, the absorbed MW energy causes internal heating due to the dielectric loss or as a result of the display rotational/conformational movement of dipoles. Unlike conventional heating that relies on slow thermal conduction and convection, MW heating allows for rapid and uniform heat transfer through radiation and direct excitation of molecular bonds. This leads to a more efficient dissolution of the polymer chains and effective polymer chain swelling, which upon cooling allows the polymer chains to adopt the lowest energy conformation, ultimately leading to the formation of well-ordered polymer aggregates.^23,24^ Noted that in the context of MW heating and conjugated polymer processing, chain swelling refers to the volumetric expansion of polymer chains due to solvent penetration or thermal/electrochemical stimuli, which alter their conformation and aggregation behavior. This phenomenon is critical in determining microstructure stability and device performance during processing.^25,26^

**Figure 1:**
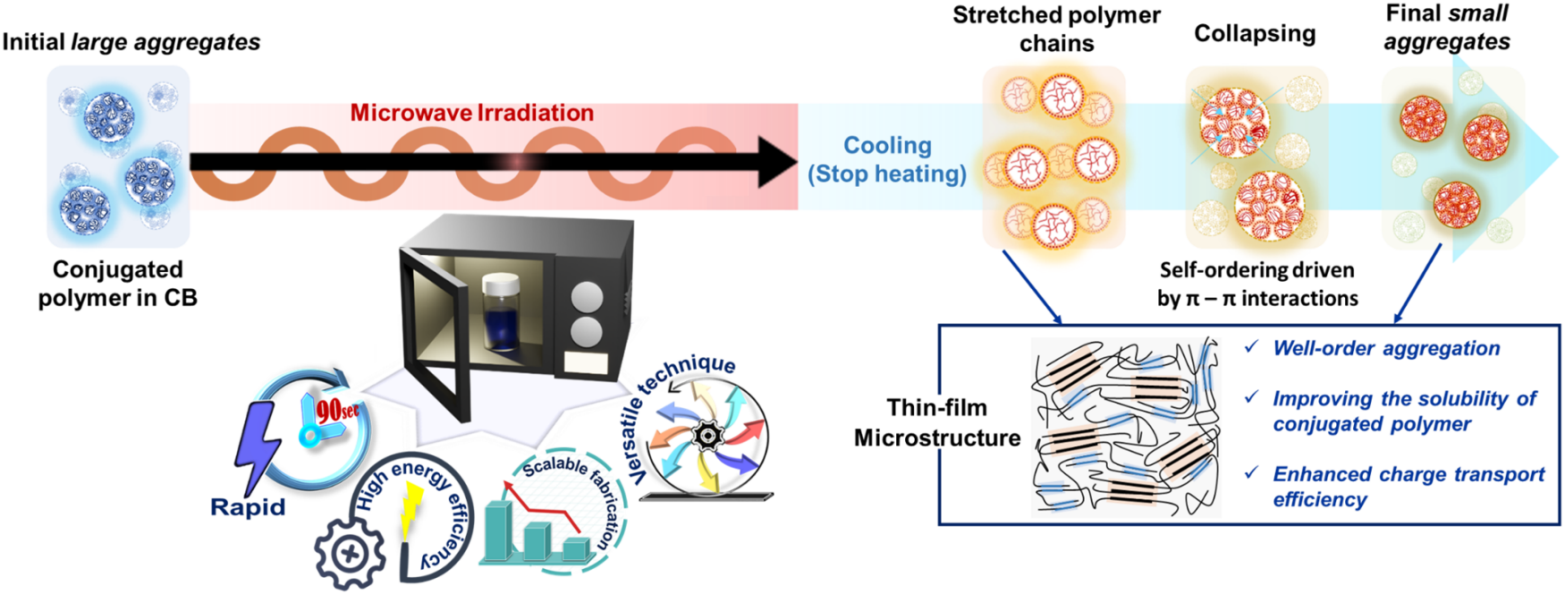
Schematic depiction of the mechanism for microstructural alteration of solution-state aggregates through the MW heating process and its advantages.

## Results and Discussion

To prove the above concept, poly­[*N,N*′-bis­(2-octyldodecyl)­naphthalene-1,4,5,8-bis­(dicarboximide)-2,6-diyl]-*alt*-5,5′(2,2′-bithiophene), well-known as N2200 or P­(NDI2OD-T2), was employed as a model conjugated polymer due to it exhibiting high electron mobilities of over 0.1 cm^2^ V^–1^ s^–1^.^27^ The strong donor–acceptor (D–A) nature and rigid π-conjugated backbone of this polymer imparts P­(NDI2OD-T2) with face-on orientation in thin films and significant aggregation in numerous organic solvents.^28^ Chlorobenzene (CB) was chosen as the solvent due to its relatively poor solvating ability against P­(NDI2OD-T2), and its slightly higher polarizability (relative to other aromatic hydrocarbons) facilitating MW heating of P­(NDI2OD-T2) in both CB and 1-chloronaphthalene (CN) at room temperature (RT). Note that as a nonpolar organochlorine compound, CN has a good affinity with P­(NDI2OD-T2). Thus, CN was selected as the control solvent for solubility comparison with CB. As shown, the P­(NDI2OD-T2) absorption spectrum revealed two spectral features: a high-energy band around 400 nm that is attributed to the π–π* transition and the second, a broad absorption band around 600–700 nm, ascribed to the charge-transfer (CT) transition between the donor and acceptor moieties along the main polymer chain.^28^ Based on the spectra, the CT absorption peak displays a bathochromic shift that is more significant than the π–π* transition. The absorption spectra in Figure S1 are consistent with the observations by Neher et al., in which P­(NDI2OD-T2) was dissolved in both good and poor solvents.^28^ Moreover, Figure S2 shows a distinctive solution color, where the solution in CN displayed a deep blue color, while the CB solution was light blue. These results seem to indicate the effect of solvent polarity on the aggregation of P­(NDI2OD-T2). Unlike CB, which only contains one benzene ring, CN is constituted of two benzene rings; thus, it can induce more π–π interactions with P­(NDI2OD-T2) than CB, leading to better solubility. Interestingly, upon the MW irradiation of the P­(NDI2OD-T2) in CB, its color transformed and has the same deep blue color as in CN, suggesting the occurrence of chain swelling.^28^ And even more, the result qualitatively demonstrated the effectiveness of MW heating in enhancing the solubility of P­(NDI2OD-T2) in a poor solvent at elevated temperatures of 80 °C. These results indicate the benefits of using MW irradiation and heating over conventional heating in activating molecular vibration and rotation, which subsequently alter the microstructural ordering of the solution-state polymer aggregates. However, the mechanisms underpinning polymer dissolution under MW radiation and subsequent aggregate formation in solution remain elusive. Thus, time-resolved *in situ* diagnostic techniques were integrated into the MW oven to shed light on the dynamic mechanism as illustrated in Figure 2a. *In situ* absorption and photoluminescence (PL) spectroscopies were employed to investigate the effectiveness of MW heating to the P­(NDI2OD-T2) solution in CB. The evolution of absorption spectra from P­(NDI2OD-T2) solution in CB upon MW heating and natural cooling are depicted in Figure 2b. The heating rate was around 1.8 °C s^–1^, and then, the solution was allowed to cool to room temperature for 5 min. Upon heating, the CT absorption peak gradually shifted from 697 nm at RT to 599 nm at 80 °C, approaching the wavelength of CT absorption in a good solvent (Figure S2). This implies that the polymer chains were well swollen by employing MW heating despite CB being a poorer solvent. As the solution naturally cooled to RT (see the dashed lines in Figure 2b), the CT absorption peak red-shifted back to the original position with noticeable higher intensity and narrower peak width, suggesting the renewed formation of aggregate polymer structures.

**Figure 2:**
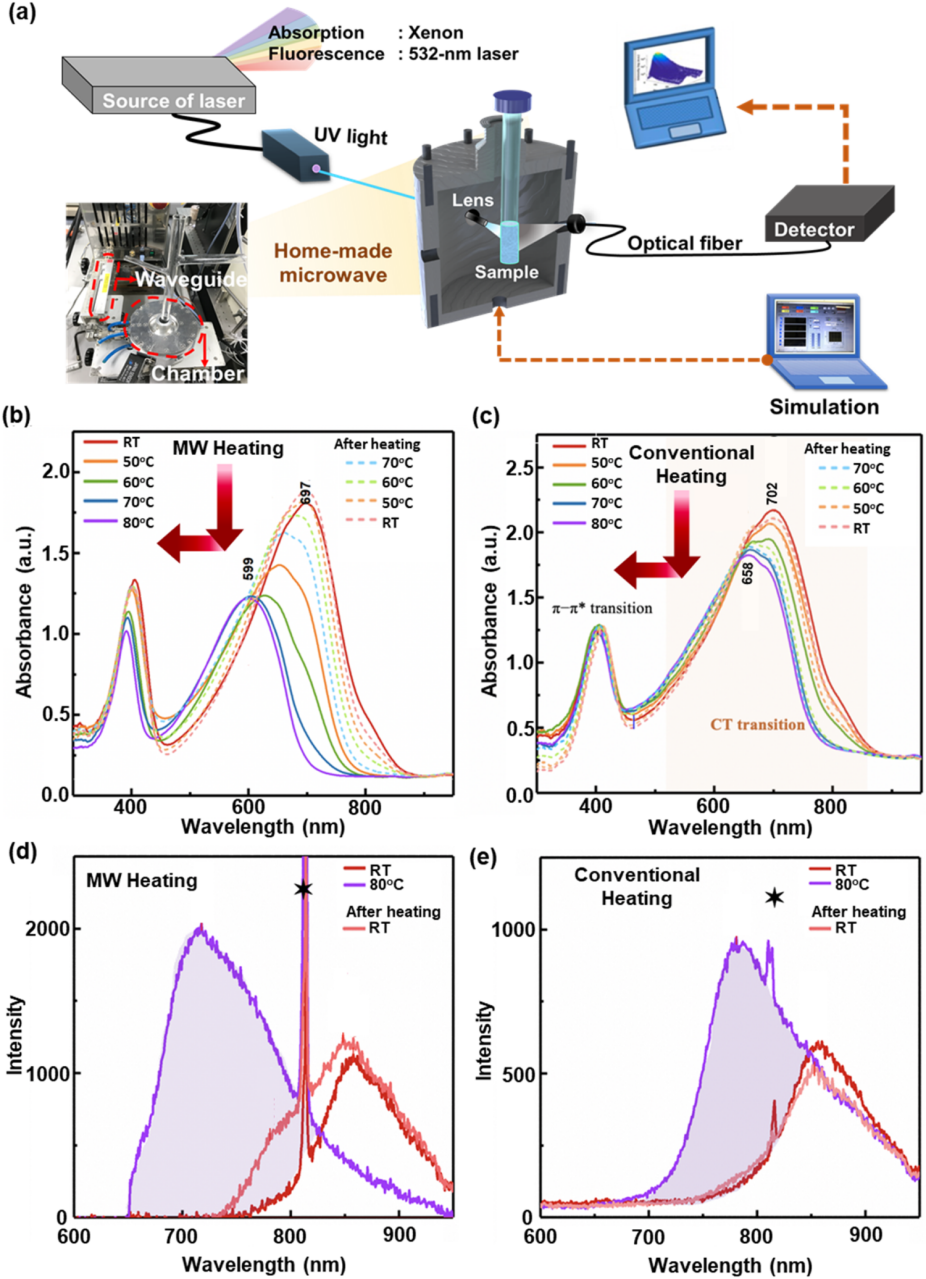
(a) Schematic of the *in situ* optical absorption/photoluminescence (PL) spectroscopies of a conjugated polymer solution during microwave heating. The time-resolved absorption spectrum of 0.05 mg mL^–1^ P­(NDI2OD-T2) in a CB solution with (b) MW heating and (c) conventional heating. The solid lines correspond to the heating process, which entailed an increase in temperature from room temperature (RT) to 80 °C, whereas the dashed lines represent the cooling process, where the temperature decreased from 70 °C to room temperature. Additionally, the red arrows indicate the blue shift peak and the decrease in absorbance as the heating temperature increases. PL spectra of 0.05 mg mL^–1^ P­(NDI2OD-T2) in CB were obtained with (d) MW heating and (e) conventional heating with an excitation wavelength of 405 nm. The peaks marked with a star are 2nd-order Rayleigh scattering from a 405 nm excitation wavelength.

To highlight the efficacy of MW heating, we compared the ultraviolet–visible (UV–vis) absorption spectra to those of a sample heated conventionally using a water bath. As depicted in Figure 2c, the observed CT absorption peak only shifted by about 41 nm (heating from RT to 80 °C), which is less than half of the shift observed under MW heating (∼98 nm). This signifies that conventional heating, relying on thermal conduction and thermal convection, may only partially swell polymer aggregates. As the aggregates rearrange from a mere partially swollen aggregate, it may not greatly alter the structural ordering of the initial aggregates in the CB. This resulted in a bathochromic shift and higher CT absorbance in the cooled solution at RT for conventional heating compared with MW heating.

Considering the feasibility of MW heating for any type of highly semicrystalline conjugated polymers,^29,30^ we also examined poly­(3-hexylthiophene) (P3HT) and poly diketopyrrolopyrrolo-thieno­[3,2-*b*]­thiophene (PDPP2T) as representative p-type semiconductor polymers. The *in situ* absorption spectrum recorded between 300 and 600 nm for P3HT revealed a slight blue shift in the π–π* transition from 515 nm at RT to 503 nm at 80 °C and a decrease in absorbance with rising MW heating temperatures (Figure S3a). The *in situ* absorption spectrum of PDPP2T is characterized by two spectral features: a high-energy peak located at 435 nm attributed to the π–π* transition of the aromatic thiophene donor segment and a low-energy peak located at 783 nm ascribed to the CT transition. As the temperature increased from RT to 80 °C, both main peaks of PDPP2T exhibited obvious blue shifts of 18 and 24 nm, with the high-energy peak shifting gradually from 435 to 417 nm and the low-energy peak shifting from 783 to 759 nm, respectively (Figure S3b). These blue shifts reflect alterations in the donor–acceptor electronic interactions, which are commonly associated with reduced polymer chain aggregation or changes in molecular packing. Through closer observation, it was found that the *in situ* absorption spectra analysis for PDPP2T showed a more pronounced blue shift at both characteristic peaks compared with P3HT (12 nm) under identical conditions. The significantly larger blue shift observed for PDPP2T compared to P3HT can be attributed to the distinct solubility profiles of the two conjugated polymers in CB. PDPP2T, which has relatively low solubility at RT, experiences enhanced solvation upon MW irradiation due to the MW-induced molecular vibrations facilitating polymer swelling by increasing segmental mobility of polymer chains and disrupting intermolecular forces through localized heating and dipolar excitation.^23,24^ Conversely, P3HT is dissolved in CB at RT with relatively high solubility and results in minimal aggregation; therefore, MW irradiation causes only subtle changes in the swollen polymer chains, resulting in a slight blue shift. Furthermore, the distinct reduction in absorbance for PDPP2T (Figure S3b) compared to P3HT (Figure S3a) emphasizes the significant effect of MW irradiation on promoting solubility in donor–acceptor conjugated polymers. This behavior aligns with established theories wherein decreased aggregation leads to diminished intermolecular electronic coupling, manifesting as lower absorbance intensity and spectral blue shift in UV–vis measurements.^28^ The consistent spectral changes induced by molecular vibrations under MW irradiation underscore the efficacy of MW irradiation as a promising strategy to address solubility challenges in other semicrystalline conjugated polymers.

Furthermore, *in situ* PL measurements were conducted to affirm the difference in chain swelling and reaggregation of the P­(NDI2OD-T2) solution in CB between MW heating and conventional heating. Figure 2d shows the PL spectra of a solution before (at RT), upon (at 80 °C), and after (at RT) MW heating. The solution before MW heating exhibited a characteristic emission at 858 nm. A notable blue shift to 718 nm upon heating was observed. For the cooled solution, the spectrum reverted to a slightly shorter wavelength, around 848 nm. A distinct broad shoulder at around 781 nm was also concurrently observed. By contrast, the conventionally heated solution displayed a significantly smaller shift (about 75 nm) compared to the MW-heated sample (about 140 nm) while keeping the same spectral feature with somewhat reduced intensity in the cooled solution, as seen in Figure 2e. The appearance of a broad shoulder (∼780 nm) in the PL spectrum of the polymer solution after MW heating was an indicator of the rearrangement of swollen polymer chains into a distinctly ordered reaggregate. At this point, we speculated that the extended polymer chains, swollen by MW heating, may follow different dynamics and, thus, substantially affect the reaggregation behavior. While both absorption and PL characteristics changed under MW heating, the same trend was not observed in the conventionally heated solutions, affirming the challenge of thermal conduction and convection to efficiently swell polymer chains.

To confirm the polymer chain dynamics under MW heating–cooling as discussed in optical analyses above, the swelling to collapsing behaviors, aggregate size, and chain conformation of polymer in solution were further investigated by *in situ* small-angle X-ray scattering (SAXS) analysis. In Figure 3a, the scattering profile of the initial solution in CB at RT exhibited high scattering intensity, especially in the lower *q* regions (<0.07 Å^–1^). This indicates that the initial solution possessed large polymer aggregates. Upon heating with MW irradiation, the scattering footage became much lower as may be attributed to the thermal-induced disruption of the strong π–π interactions between polymer chains, reducing the polymer aggregates in solution.^31,32^ Intriguingly, the scattering profile of the solution in CB upon MW heating is similar to that of the solution in CN at RT (see Figure S4a,b), suggesting the progression of swelling chains upon heating. However, a clear hump in the high *q* values (>0.1 Å^–1^, marked with a red arrow in Figure S4a) for solution in CN at RT indicated the existence of long-range interactions of stacked interchains which did not occur in solutions of CB heated under MW irradiation.^33^ This discrepancy further corroborates the thermally induced disruption effect in polymer solutions. Compared to MW heating, the polymer aggregations remained in solution as shown by a relatively higher scattering intensity profile in the lower *q* ranges seen in Figure 3b. These results are consistent with the *in situ* optical spectra of partially swollen aggregates. Even though both methods heated the solution to the same temperature (∼85 °C), thermal-induced disruption using MW heating can effectively break down the polymer aggregates, not only by molecular vibration but also by molecular rotation, leveraging the conformational degree of freedom in a polymer chain.

**Figure 3:**
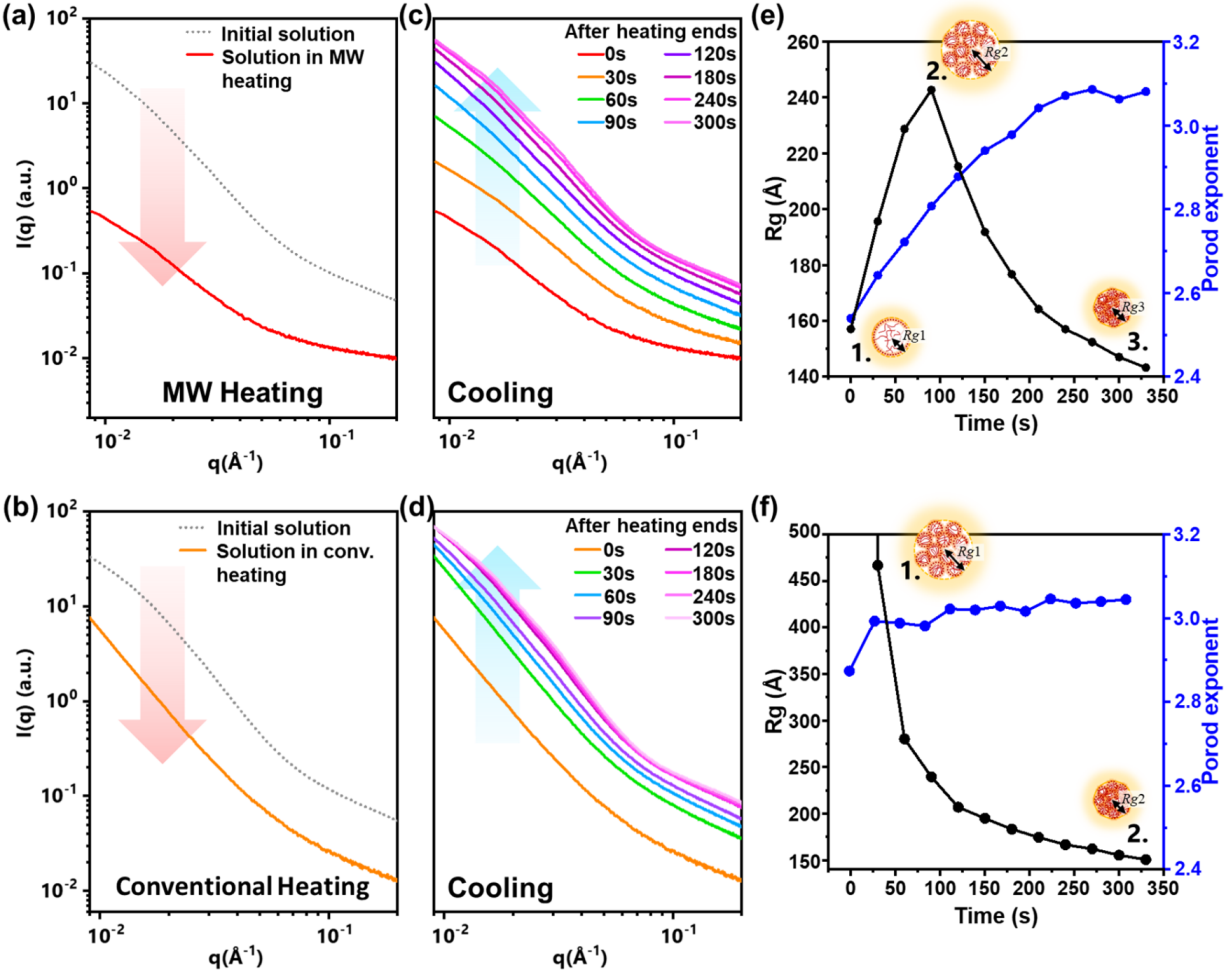
SAXS profiles for 8 mg mL^–1^ of P­(NDI2OD-T2) in CB and the fitting parameters. (a) SAXS profiles of the initial solution under MW heating (1200 W, 30 s, at 85 °C) and (b) conventional heating using water bathing at 85 °C. The SAXS profiles of cooled solutions were collected at times of 0, 30, 60, 90, 120, 180, 240, and 300 s after ceasing (c) the MW heating and (d) the conventional heating. The radius of gyration (*R*_g_) and the Porod exponent values are based on the fitting results of the cooling process after the (e) MW heating and (f) conventional heating.

Additionally, the reaggregation behaviors were observed through a cooling process as depicted in Figure 3c,d. After the heating process of both methods was ceased, the scattering intensities gradually increased over cooling time (from 0 to 300 s) and ended with a relatively higher scattering intensity than their initial solutions. This suggests that the number of aggregates in solution after the heating process might increase. Nevertheless, the reaggregation behaviors after MW heating would be different from conventional heating since it proved to be effectively swelling the polymer aggregates. To get a deeper insight, the scattering profiles of cooling processes were fitted by a model that describes the polymer chain conformations with excluded volume.^34^ The model is parametrized by the excluded volume parameter ν, which is related to the Porod exponent m as ν = 1/m. Additionally, the statistical segment length of the polymer chain is denoted by *a*, and *n* is the degree of polymerization. The form factor that describes the scattering intensity of a particle as a function of its size and shape was expressed with an integral form as follows

1 $$P\left(q\right)=2\int_{0}^{1}dx\left(1-x\right)\exp\left[-\frac{q^{2}a^{2}}{6}n^{2v}x^{2v}\right]$$

The integral equation above is then derived as expressed in the formula below

2 $$P\left(q\right)=\frac{1}{vU^{1/2v}}\gamma \left(\frac{1}{2v},U\right)-\frac{1}{vU^{1/v}}\gamma \left(\frac{1}{v},U\right)$$

where γ is the incomplete γ function.

3 $$\gamma \left(x,U\right)=\int_{0}^{U}dt\;\exp\left(-t\right)t^{x-1}$$

The variable *U* is given in the form of the scattering vector *q* as

4 $$U=\frac{q^{2}a^{2}n^{2v}}{6}=\frac{q^{2}{R_{g}}^{2}\left(2v+1\right)\left(2v+2\right)}{6}$$

Subsequently, the radius of gyration (*R*_g_) could be defined as stated below

5 $$R_{g}^{2}=\frac{a^{2}n^{2v}}{\left(2v+1\right)\left(2v+2\right)}$$

The above model accounts for the form factor equation that is applicable only within the mass fractal range, noting that fully swollen chains in a good solvent will have a Porod exponent of 5/3 and then for the partially swollen chains (in an intermediate solvent) may have a Porod exponent of 2, whereas the collapsed chains or aggregates in a poor solvent have a Porod exponent of around 3.

The model given above is well-fitted with the scattering profiles over the cooling time presented in Figure S5. The deduced *R*_g_ value and the Porod exponent from the scattering of cooling profiles after the MW heating is stopped are plotted in Figure 3e. At 0 s, the small *R*_g_ value around 157 Å indicated that the large aggregates were broken into some smaller aggregates and eventually transformed into swollen chains with the Porod exponent of about 2.54 confirming the mass fractal value. The *R*_g_ value then increased to 243 Å at 90 s with the Porod exponent moved up to 2.81. This may imply that the swollen chains started to collapse, reordering, and reaggregating due to the loss of thermal-induced disruption effect and driven by the strong π–π interactions, which agree with the *in situ* photophysical properties. As the cooling time continued for over 300 s, the smaller polymer aggregates with the *R*_g_ value of about 147 Å, confirmed by the Porod exponent of close to 3.06, were formed. In stark contrast, there was no collapsed transition observed from conventional heating. As depicted in Figure 3f, from 0 to over 300 s after ceasing the conventional heating, the large aggregates directly decreased in size from over 450 Å to as small as 155 Å. The conventional heating may not be able to swell the initial solution aggregates, but merely expand the polymer aggregates without disentangling the polymer chains. The small change in the estimated Porod exponent ranging from 2.87 (at 0 s) to 3.04 (at over 300 s) as the cooling time progresses may affirm the polymer in solution remained as aggregates. This is also corroborated by the small shift that occurred in both optical absorption and PL. To simplify the above explanation, the swelling-collapsing dynamics mechanism of the conjugated polymer into ordered aggregates under MW heating is summarized schematically in Figure 1

### Thin Film Morphology of P­(NDI2OD-T2)

Optical and scattering data demonstrated the conspicuous features of P­(NDI2OD-T2) solution-state aggregations after MW heating. We employed spin-coating of the polymer solution to further explore the microstructure alteration of the P­(NDI2OD-T2) films. An atomic force microscopy (AFM) phase image in Figure 4a showed the surface topology structure of the as-cast spin-coated film from a P­(NDI2OD-T2) solution with MW heating. The film revealed a clear picture of fine- and dense-fibrillar microstructures with smaller domain sizes, no preferential orientation, and root-mean-square (RMS) roughness of 0.477 nm. Oppositely, for the as-cast spin-coated film from a solution without MW heating, the AFM image presented shorter fibrils with a wider range of diameters, as illustrated in Figure 4b. Although it is well-known that post-thermal annealing can improve the microstructure of the as-cast film, the post-annealed film from Figure 4b, shown in Figure 4c, produced fibrillar microstructures that were not as fine and continuous as with MW heating. Instead, the obvious larger grain structures may suggest there are many grain boundaries available as a source of deep traps that could lower the charge transport efficiency.^15^ These results are also supported by the increasing surface roughness from 0.493 to 0.591 nm. The average fibril diameters of the P­(NDI2OD-T2) films can also be evaluated through the full width at half-maximum (FWHM) of the line profiles from the entire AFM phase image *x*-axis,^35^ which were extracted from the two white direction lines in Figure S6a–c. The average fibril diameter obtained is also consistent with the aforementioned statement for P­(NDI2OD-T2) films processed with MW heating, as-cast and post-annealing treatments are 14.2 ± 3.6, 15.4 ± 5.7, and 18.5 ± 5.4 nm, respectively, as shown in Figure 4d–f.

**Figure 4:**
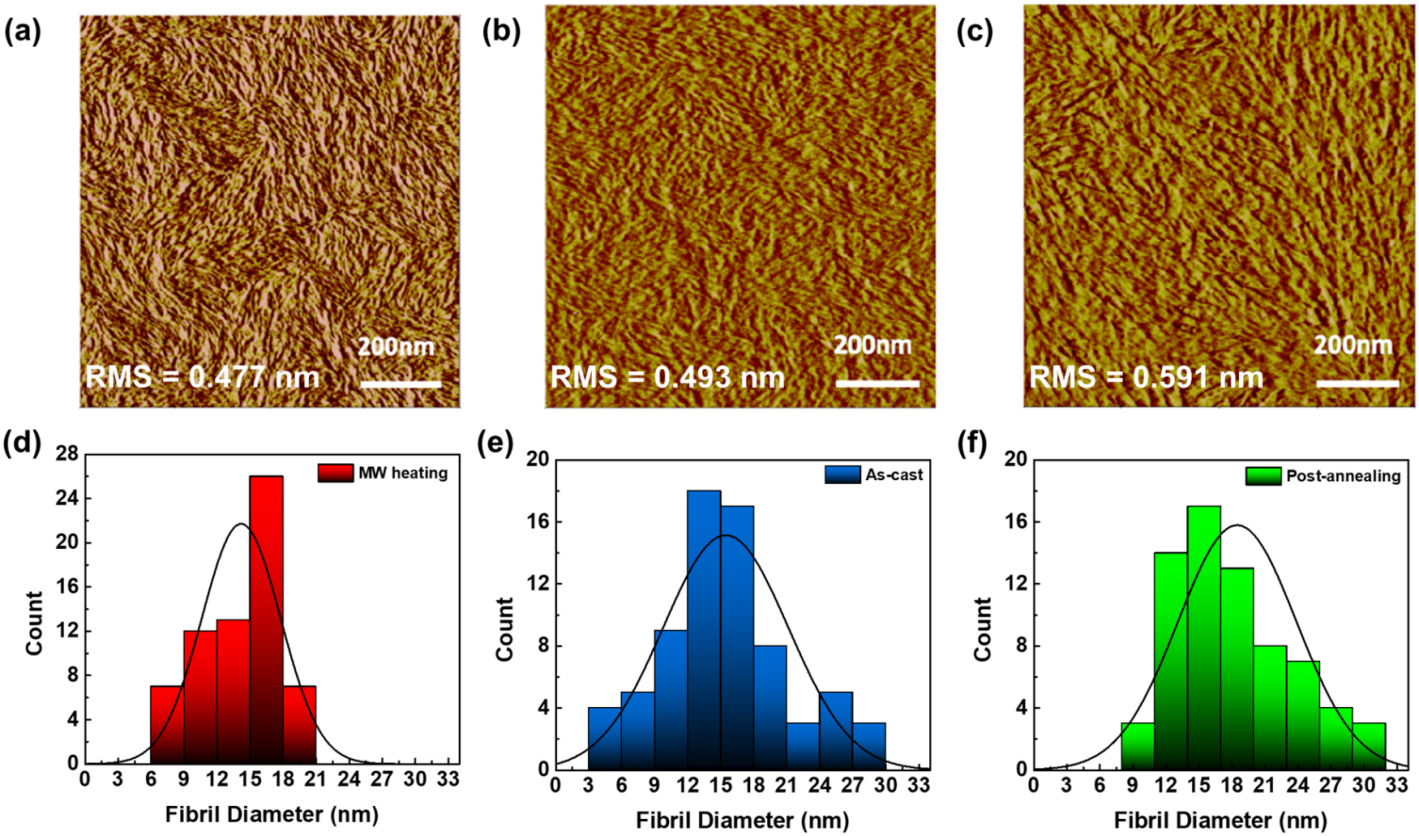
AFM phase images and their corresponding fibril diameter distribution histograms of spin-coated P­(NDI2OD-T2) films: (a, d) As-cast film from a solution with MW heating, (b, e) as-cast film from a solution without MW heating, and (c, f) post-annealed film from a solution without MW heating at 170 °C for 1 h.

Aside from topological structure analysis, the molecular ordering of polymer films was also investigated by measuring the grazing-incidence with wide-angle X-ray scattering (GI-WAXS) on P­(NDI2OD-T2) films. Figure 5a showcases the diffraction pattern of an as-cast polymer film from a solution with MW heating. The noticeable X-ray reflections from (*h00*) and (*00l*) diffraction planes, referred to as lamella stacking and chain backbone repeating unit, respectively, were observed along the in-plane (*q*_*xy*_) direction. The spacings of both lamella structures and chain backbone repeating units were found to be 25.12 and 13.63 Å, respectively, which gave the values in agreement with other reported microstructures of P­(NDI2OD-T2) films in the literature.^27,33^ Whereas a defined (*0k0*) diffraction arc or π–π stacking in the out-of-plane (*q*_*z*_) direction indicated the molecular packing motif of P­(NDI2OD-T2) film adopting face-on orientation (see Figure 5e), with a π–π distance of about 4.00 Å as tabulated in Table 1. Similar diffraction patterns were recorded for the as-cast film from a solution without MW heating, however, with much fewer diffraction traces, probably due to the disordered packing of large aggregates as presented in Figure 5b. After the post-annealing process, the polymer film in Figure 5b evolved with a better-ordered microstructure as the diffraction patterns and showed a rise in intensity along with a noticeable π–π reflection plane in the *q*_*z*_ direction as seen in Figure 5c. Moreover, P­(NDI2OD-T2) film annealed at 170 °C predominantly adopts the thermodynamically stable form I polymorph, characterized by the π–π stacking distance of 3.99 Å and lamellar spacing of 25.12 Å, consistent with previous reports in the literature for P­(NDI2OD-T2).^36–38^ Using these GI-WAXS studies, we confirmed that the as-cast polymer film from a solution with MW heating can generate a highly crystalline film, even when compared to post-annealing processes on films from conventionally heated solutions. The decreased full width at half maxima (FWHM) values of the (*0k0*) plane in the as-cast polymer film with MW heating in solution, observed in Figure 5d and Table S1, further supported that the crystalline domains are larger than those formed by the post-annealing process. The crystalline coherence lengths of P­(NDI2OD-T2) films calculated using the Scherrer equation were also evaluated under different processing conditions (Table S1). The as-cast thin film prepared via MW heating for 20 s in solution exhibited a coherence length of 13.2 nm, higher than the 11.7 nm coherence length observed in films formed by post-annealing at 170 °C for 1 h, implying a lower density of grain boundaries in the MW-heated film, as fewer interfaces exist between misoriented domains. Thus, the advantage of MW irradiation is confirmed as it facilitates rapid and uniform energy distribution at the molecular level, promoting improved molecular alignment and packing in thin films. Consequently, the as-cast thin film prepared via MW heating in solution results in better crystallinity and larger coherence lengths compared to those of conventional thermal annealing.

**Figure 5:**
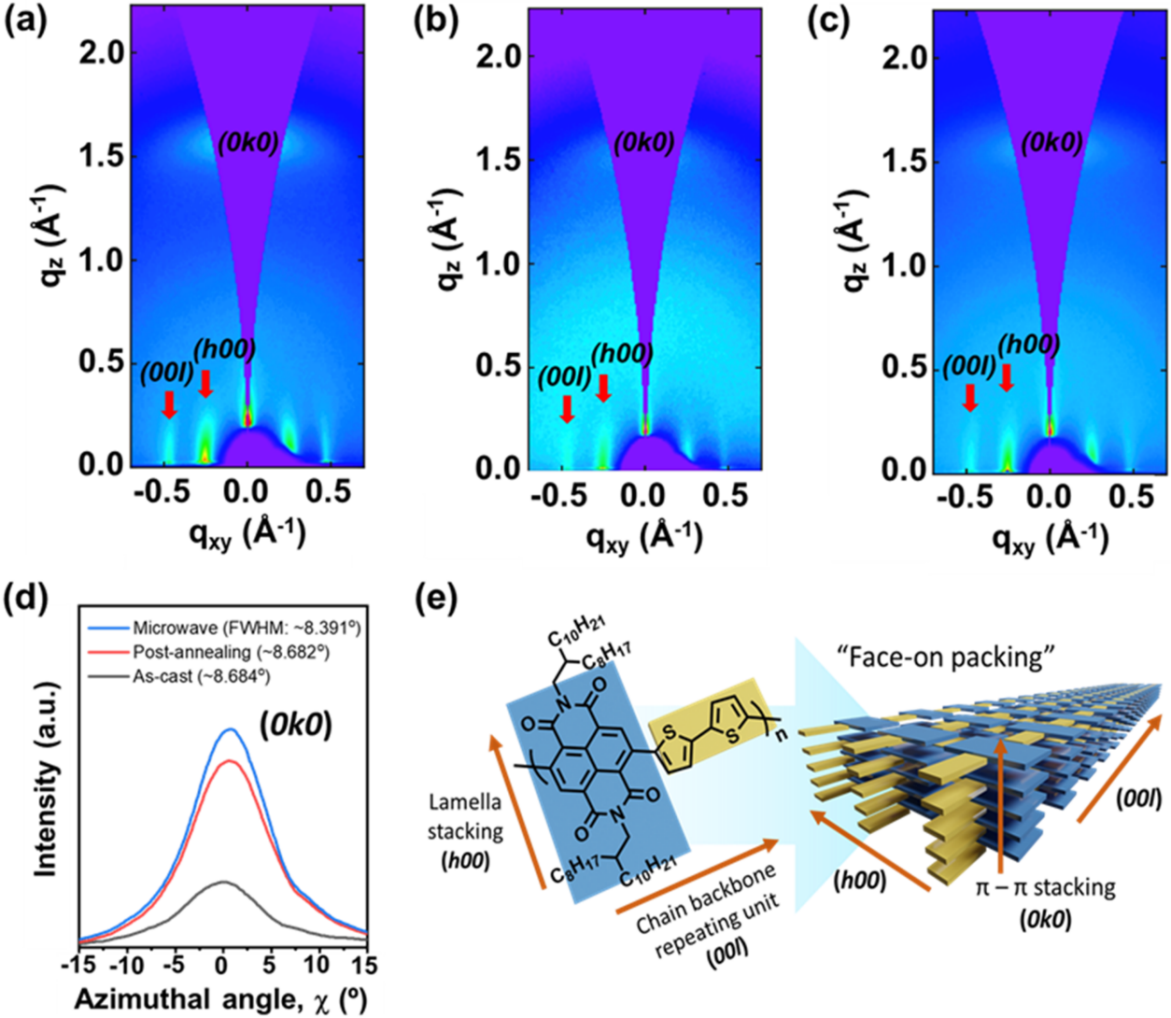
GI-WAXS patterns of P­(NDI2OD-T2) films. (a) As-cast film from a solution with MW heating for 20 s, (b) as-cast film from a solution without MW heating, and (c) post-annealed film from a solution without MW heating at 170 °C for 1 h. (d) Azimuthal angle dependence of the (*0k0*) peak intensity. (e) Schematic illustration of the molecular packing in P­(NDI2OD-T2) films, highlighting lamellar stacking (h00), π–π stacking (*0k0*), and the face-on orientation relative to the substrate surface.

**Table 1:** *q* Values and *d*-Spacing Parameters Determined from GI-WAXS Patterns

	*q* (Å^–1^)			*d*-spacing (Å)		
thin film condition	(*h00*)	(*00l*)	(*0k0*) π–π stacking	(*h00*)	(*00l*)	(*0k0*) π–π stacking
MW	0.25	0.46	1.57	25.12	13.63	4.00
as-cast	0.26	0.46	1.52	24.47	13.63	4.14
post-annealing	0.25	0.47	1.58	25.12	13.41	3.99

### Organic Field-Effect Transistor Performance

To understand the effect of microstructure alteration of solution-state aggregates via MW heating on the molecular packing of thin film, we examined the electrical characteristics of an organic field-effect transistor (OFET) using a P­(NDI2OD-T2) active layer prepared from a solution with MW heating, as illustrated in Figure 6a. The transfer curves and output curves of OFETs with different processing of P­(NDI2OD-T2) active layers are displayed in Figures 6b and S7, respectively. As shown, the OFET using the P­(NDI2OD-T2) active layer from a solution with MW heating exhibited typical n-type characteristics along with higher output drain current modulation and saturated electron charge mobility (μ_e_ saturation) of about 0.45 cm^2^ V^–1^ s^–1^. Among other processes (see Table 2), high carrier mobility, as high as 3.6-fold enhancement, can be achieved compared to the prepared as-cast active layer without MW heating. This may have originated from the ordered molecular packing of small aggregates that formed through the collapsing process of extended/stretched conjugated polymer chains due to MW irradiation activating molecular vibration and rotation, effectively swelling the polymer aggregates, as suggested by scattering profile data. The highly ordered small aggregates in solution could provide better microstructure packing during film formation at segmental levels, which was proven by GI-WAXS data. On top of this, the carrier mobility of P­(NDI2OD-T2) from a solution with MW heating also remained higher than that of the post-annealed film from a solution without MW heating. Figure 6c illustrates the plausible microstructural differences after film formation. The as-cast film from a solution without MW heating formed mostly amorphous or disordered polymer regimes that could be considered as deep traps for the charge carriers and thus limited the charge transport. In contrast, the as-cast film from a solution with MW heating replaced the amorphous regimes with a highly ordered small aggregate that filled in within the crystalline regions in polymer films. This way, the highly ordered small aggregates acted as a short conductive pathway to connect from one crystalline region to another via multiple trapping and release mechanisms, rendering efficient intermolecular charge transfer.^15,16^ Such a phenomenon is also applicable to other conjugated polymer derivatives including poly­(3-hexylthiophene) (P3HT), polydiketopyrrolopyrrole-thieno­[3,2-*b*]­thiophene (PDPP2T), and poly­(isoindigo-bithiophene)-based donor–acceptor copolymer P­(IID-BT), which exhibit carrier mobility value trend consistent with P­(NDI2OD-T2) (see Figures S8–S10 and Table S2). For example, P3HT prepared from a solution with MW heating generates the highest charge carrier mobility value of 0.10 cm^2^ V^–1^ s^–1^ while P3HT prepared from as-cast generates the lowest charge carrier mobility value of 0.06 cm^2^ V^–1^ s^–1^. These results emphasize the strategic implementation of MW heating that facilitates enhanced solubility of conjugated polymers and well-order aggregation, leading to optimized electrical properties (Figure 1). The consistency of this trend across different conjugated polymer systems underscores the importance of processing conditions in optimizing the electronic properties of organic semiconductors.

**Figure 6:**
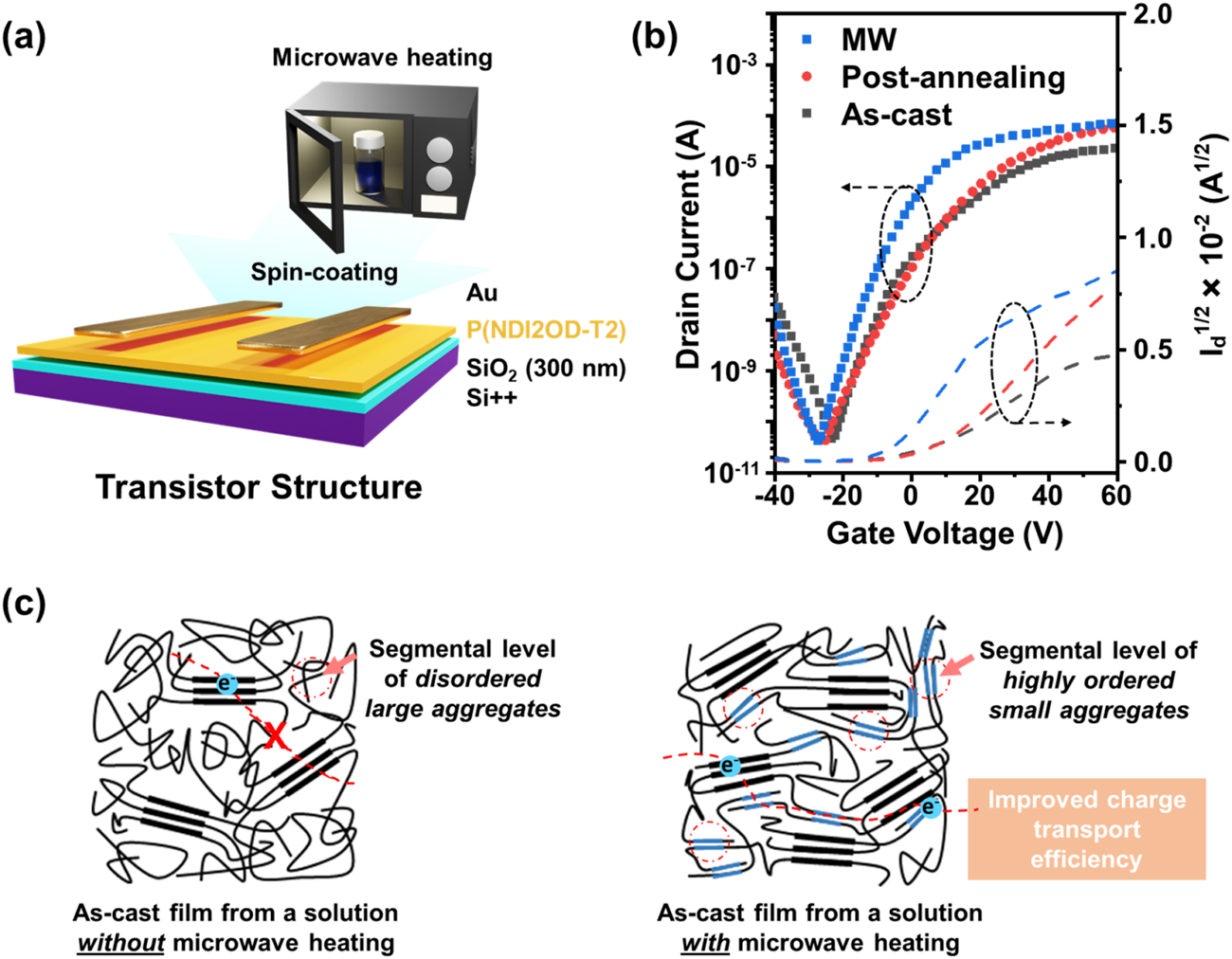
(a) OFET structure and fabrication. (b) Electrical test of P­(NDI2OD-T2) OFETs prepared from a solution with MW heating, as-cast film without MW heating, and post-annealing of film from a solution without MW heating at 170 °C for 1 h. The measurement was set up at *V*_ds_ (source-drain voltage) of 60 V, and *V*_g_ (gate voltage) was swept from −20 to 60 V. (c) Illustration of microstructural alteration of P­(NDI2OD-T2) films used in OFETs.

**Table 2:** Electrical Characteristics of P­(NDI2OD-T2) OFETs

process condition	average field-effect mobility (cm^2^ V^–1^ s^–1^)	*V*_th_ (V)	on/off ratio
MW	4.47 × 10^–1^	–6.0	1.30 × 10^6^
post-annealing	2.47 × 10^–1^	–1.0	1.28 × 10^6^
as-cast	1.24 × 10^–1^	–5.1	1.05 × 10^6^

## Conclusions

In short, these findings demonstrated a versatile and facile process, as well as rapid operando with high-efficiency energy transfer conversion using MW heating to alter the solution-state aggregation of polymer solution. This offered well-ordered molecular packing in the thin film form for scalable solution-processed conjugated polymers. Notably, the improved microstructure of thin films was owed to the formation of self-ordering small aggregates at segmental levels from extended polymer chains after ceasing MW-activated molecular vibration and rotation. Notably, MW heating can achieve better well-ordered aggregates through enhanced polymer swelling compared with the disordered microstructure of conventional heating. As a result, the transistor performance with the P­(NDI2OD-T2) active layer prepared from a solution with MW heating possessed saturated electron carrier mobility with 3.6-fold improvement compared to films from solutions without MW heating. This study also presents a reliable and feasible processing method for use on other polymer conjugate derivatives.

## Experimental Section

### Materials

Poly­[*N*,*N*′-bis­(2-octyldodecyl)­naphthalene-1,4,5,8-bis­(dicarboximide)-2,6-diyl]-*alt*-5,5′(2,2′bithiophene) P­(NDI2OD-T2) with a weight-average molecular weight (*M*_w_) of 290 kDa, a number-average molecular weight (*M_n_*) of 150 kDa, and a polydispersity index (PDI) of 1.93 was purchased from Ossila Ltd. The *M_n_*, *M*_w_, and PDI values obtained from the gel permeation chromatography (GPC) measurements (Figure S11) were consistent with the specifications provided by the supplier. In addition, the comparison of GPC analysis results for P­(NDI2OD-T2) with and without exposure to MW irradiation revealed identical molecular weight distributions, indicating that the polymer chain characteristics remained unchanged with the MW treatment. Poly­(3-hexylthiophene) (P3HT) [*M*_w_ = 131.1 kDa, *M_n_* = 57 kDa, PDI = 2.3, Regioregularity = 91%] was purchased from UniRegion Bio-Tech (Lot# BLS25 34). Polydiketopyrrolopyrrole-thieno­[3,2-*b*]­thiophene (PDPP2T) [*M*_w_ = 138 kDa, *M_n_* = 42 kDa, PDI = 3.3] and Poly­(isoindigo-bithiophene)-based donor–acceptor copolymer P­(IID-BT) [*M*_w_ = 48 kDa, *M_n_* = 22 kDa, PDI = 2.2] were provided by Simon Rondeau-Gagné group, Canada. Toluene, chlorobenzene (CB), and 1-chloronaphthalene (CN) were purchased from Sigma-Aldrich. All chemicals were used as received.

### Fabrications

Bottom-gate top-contact OFET devices were constructed by following previously reported procedures. The source and drain electrodes were 40 nm thick Au, the dielectric layer was 300 nm thick SiO_2_, and the gate electrode was a highly doped silicon substrate that was treated with self-assembled monolayer of *n*-octadecyltrimethoxysilane (OMTS). The semiconductive layer was prepared by spin-coating using P­(NDI2OD-T2)/CB (C_6_H_5_C_l3_) (5 mg mL^–1^) or P3HT/CB (8.5 mg mL^–1^) or P­(DPP2T)/CB (5 mg mL^–1^) or P­(IID-BT)/CB (5 mg mL^–1^) solution at 1000 rpm for 60 s inside an N_2_-filled glovebox. The preparation of the semiconductive layer was differentiated based on three distinct processing methods, including 700 W MW heating for 20 s, as-cast, and post-annealing at 170 °C for 1 h in solution prior to film deposition. Note that the annealing temperature of 170 °C was selected based on the previous study, demonstrating effective enhancement of molecular order and charge transport properties in P­(NDI2OD-T2) without causing thermal degradation.^39^ These *I*_ds_ *vs* *V*_gs_ curves were typical of polymer FETs that have been prepared and subsequently measured at room temperature in a glovebox. The charge carrier mobility (μ) was calculated as shown in eq 6. The effect of thermal annealing on μ was inferred from the standard FET relation

6 $$I_{\mathrm{ds}}=\frac{W}{2L}\mu_{\mathrm{sat}}C_{i}{\left(V_{G}-V_{\mathrm{th}}\right)}^{2}$$

Where *W* (1000 μm) is the channel width, and *L* (50 μm) is the channel length of the devices.

The field-effect mobility values in our study were calculated from the linear regime by using a standard metal-oxide semiconductor field-effect transistor (MOSFET) model. This approach employs the linear-regime equation, where mobility is derived from the slope of the drain current versus the gate voltage transfer curve. To ensure statistical reliability, the mobility values were obtained from five measurements per device.

### Apparatus and Characterizations

The electrical properties of OFET were measured by a Keithley 4200-SCS semiconductor parameter analyzer at room temperature in a completely dark and inert N_2_-filled glovebox. The UV–vis absorption and photoluminescence (PL) spectra at variable temperatures by conventional heating were recorded on V-670 and F-7100 spectrophotometers (Hitachi High-Tech Corp., Tokyo) equipped with a temperature controller (STR-707, Hitachi High-Tech Corp.), respectively. The absorption and PL spectra at variable temperatures by MW heating were recorded on a home-built measurement system consisting of a MW cavity resonator designed for a 10 mmϕ cylindrical tube (frequency: 2.37–2.40 GHz, output power: 12 W), a white-light source (AQ4305, Ando Electric, Co. Ltd., Tokyo, Japan), an ultraviolet LED at 405 nm (M405L3, Thorlabs Inc.), and a photonic multichannel analyzer (PMA-12, Hamamatsu Photonics Corp. Hamamatsu, Japan). The duration time for a temperature rise from 22 to 80 °C by the MW cavity resonator was approximately 33 s. The frequency of 2.4 GHz corresponds to a wavelength of 125 mm.

Small-angle X-ray scattering (SAXS) and grazing-incidence wide-angle X-ray scattering (GI-WAXS) measurements were conducted at TPS25A1 and TLS BL13A, respectively, at the National Synchrotron Radiation Research Center (NSRRC), Taiwan. The atomic force microscopy (AFM) was measured using a multimode three-dimensional (3D) scanning probe microscope (SPM) (digital instruments) operating in tapping mode with a silicon tip.

## Supplementary Material



## Notes

UV–vis analysis; SAXS profiles; AFM 2D phase images along with their corresponding line profiles obtained from section analysis; output curves; transfer curves; and GPC measurements for PNDI2OD-T2; absorption spectra of P­(NDI2OD-T2) solution in CB and CN recorded at RT at a concentration of 0.05 mg mL^–1^; the color changes of a 0.12 mg mL^–1^ P­(NDI2OD-T2) solution under three distinct conditions (PDF)
