# Supplementary material for: Altering the Microstructure of Conjugated Polymers in Solution via Microwave Irradiation
Source: Macromolecules. 2025 Jul 1;58(14):7605–15. doi: 10.1021/acs.macromol.5c00942 (PMC12288072; doi:10.1021/acs.macromol.5c00942)
Supplement: Supplementary file 1 [file ma5c00942_si_001.pdf]

Supporting Information

Altering the Microstructure of Conjugated Polymers in Solution via Microwave Irradiation

Chia-Chun Lin,‡[1] Suhendro Purbo Prakoso,‡[1,2] Livy Laysandra,[1] Ming-Hao Chang,[1] Hai-I Jo,[1] Yen-Ting Li,[1] Kai-Lin Chen,[1] Chun-Yu Chen,[3] Lewis M. Cowen,[4] Eisuke Fujiwara,[5] Audithya Nyayachavadi,[6] Simon Rondeau-Gagné,[6] Bob C. Schroeder,[4] Shinji Ando,[5] Wei-Tsung Chuang,[3] Jhih-Min Lin,*[3] and Yu-Cheng Chiu*[1,7,8]

[1] Department of Chemical Engineering, National Taiwan University of Science and Technology, No.43, Sec. 4, Keelung Rd., Da'an Dist., Taipei City 10607, Taiwan.

[2] Advanced Institute for Materials Research, Tohoku University, 2 Chome-1-1 Katahira, Aoba Ward, Sendai, Miyagi 980-8577, Japan.

[3] National Synchrotron Radiation Research Center, 101 Hsin-Ann Road, Hsinchu Science Park, Hsinchu 30076, Taiwan.

[4] Department of Chemistry, University College London, 20 Gordon Street, London, WC1H 0AJ, United Kingdom.

[5] Department of Chemical Science and Engineering, Institute of Science Tokyo, Ookayama 2-12-1-E4-5, Meguro-ku, Tokyo, 152-8552, Japan.

[6] Department of Chemistry and Biochemistry, University of Windsor, Windsor, ON N9B 3P4, Canada.

[7] Sustainable Electrochemical Energy Development Center, National Taiwan University of Science and Technology, Taipei City 10607, Taiwan.

[8] Advanced Research Center for Green Materials Science and Technology, National Taiwan University, Taipei 10617, Taiwan.

Corresponding author: lin.jm@nsrrc.org.tw; ycchiu@mail.ntust.edu.tw

‡ These authors contributed equally to this work.

* Corresponding author

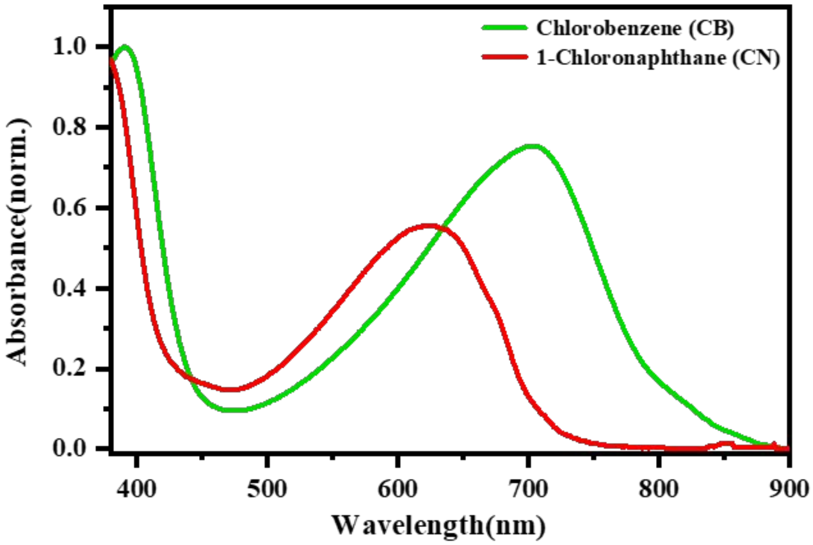

Figure S1. Absorption spectra of P(NDI2OD-T2) solution in CB and CN recorded at RT at a concentration of 0.05 mg mL^{-1}.

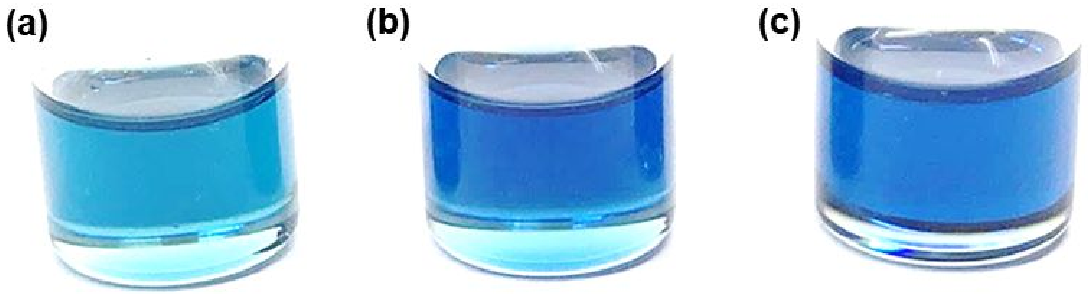

Figure S2. The color changes of a 0.12 mg mL^{-1} P(NDI2OD-T2) solution under three distinct conditions: (a) dissolved in CB at RT, (b) after microwave heating, and (c) dissolved in CN.

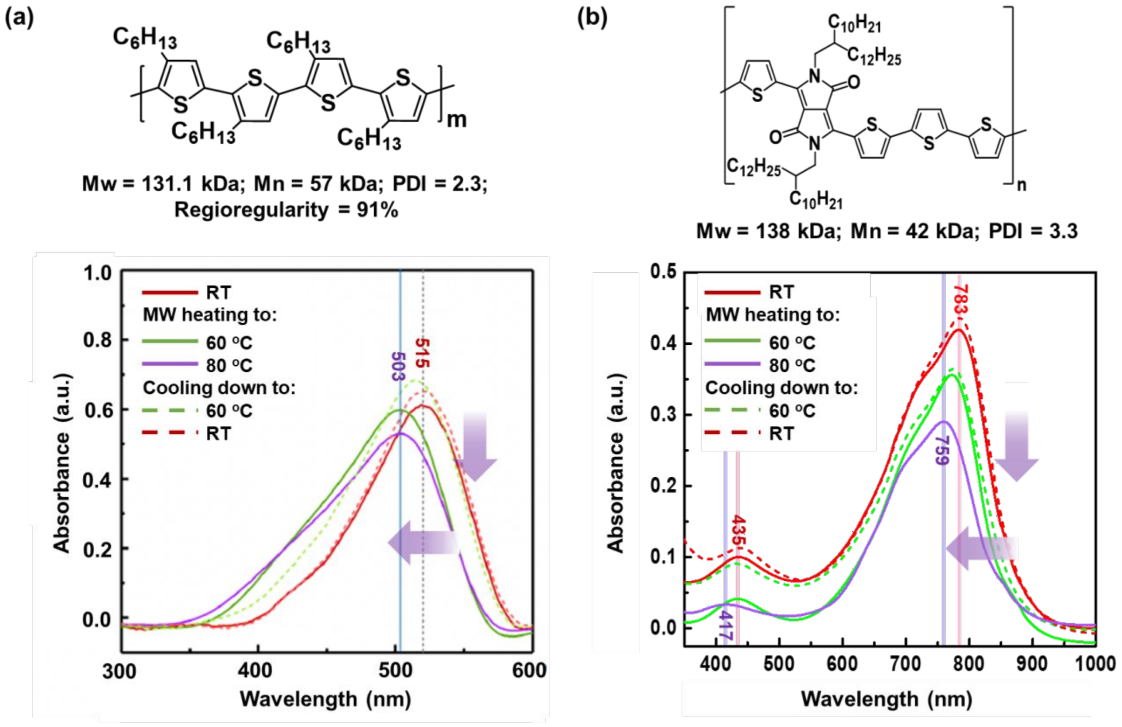

Figure S3. Chemical structures of the conjugated polymers and their corresponding *in situ* absorption spectrum results of (a) 0.0008 mg mL^{-1} P3HT and (b) 0.01 mg mL^{-1} PDPP2T in CB at different temperatures using MW irradiation.

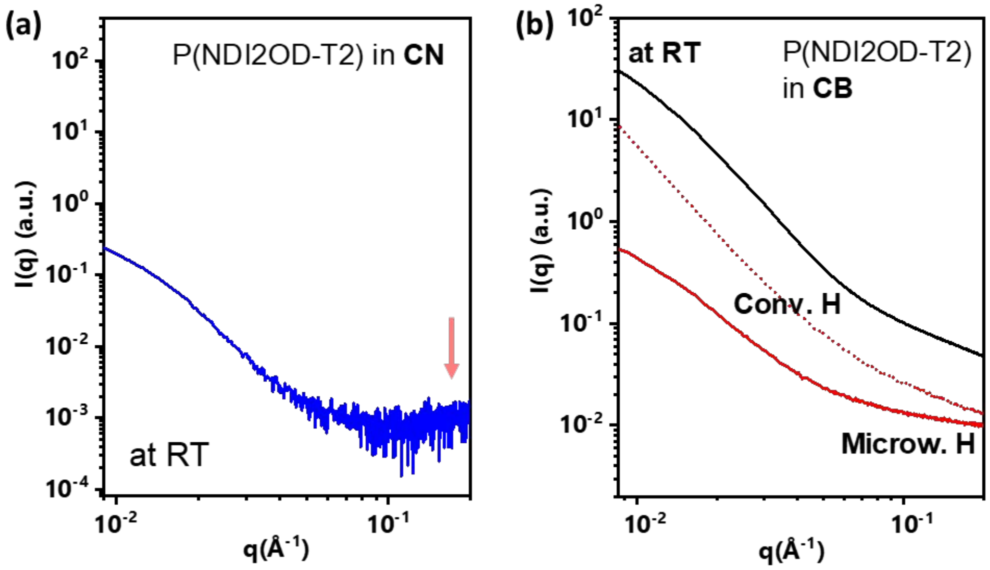

Figure S4. SAXS profiles were recorded for (a) PNDI2OD-T2 solution in CN with concentration of 8 mg mL^{-1} at RT; A red arrow indicates a hump in the q ranges over 0.1 $Å^{-1}$. (b) SAXS profiles of PNDI2OD-T2 solution in CB at RT and elevated temperature (~85 °C) under conventional heating (Conv. H) and microwave heating (Microw. H).

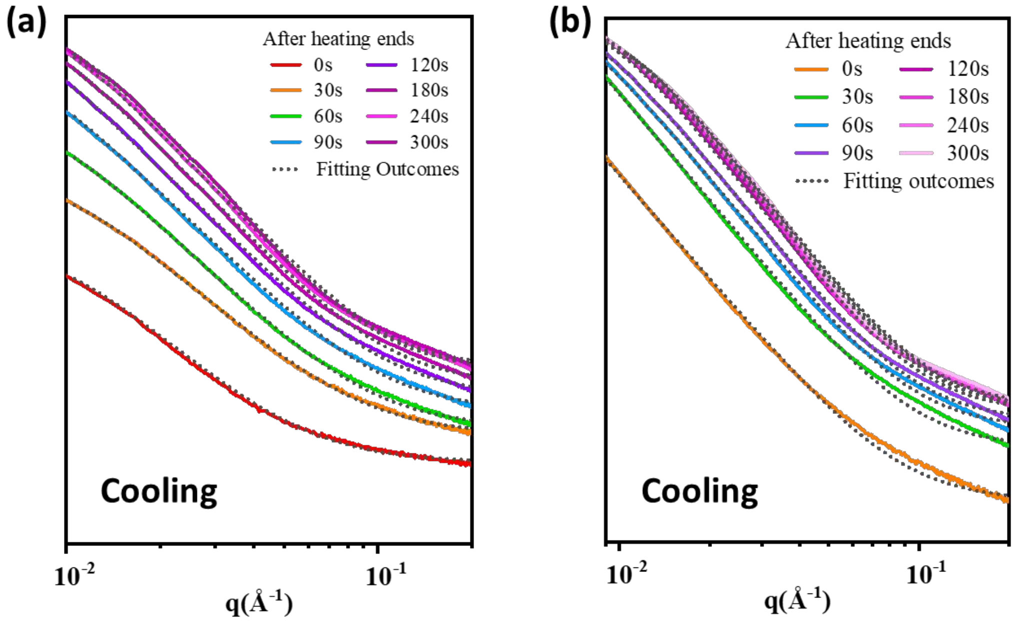

Figure S5. The fitting results of SAXS profiles from cooling processes at a time of 0, 30, 60, 90, 120, 180, 240, and 300 s. (a) Cooling processes from a solution after microwave heating and (b) after conventional heating. Dashed line denoted the fitting curves.

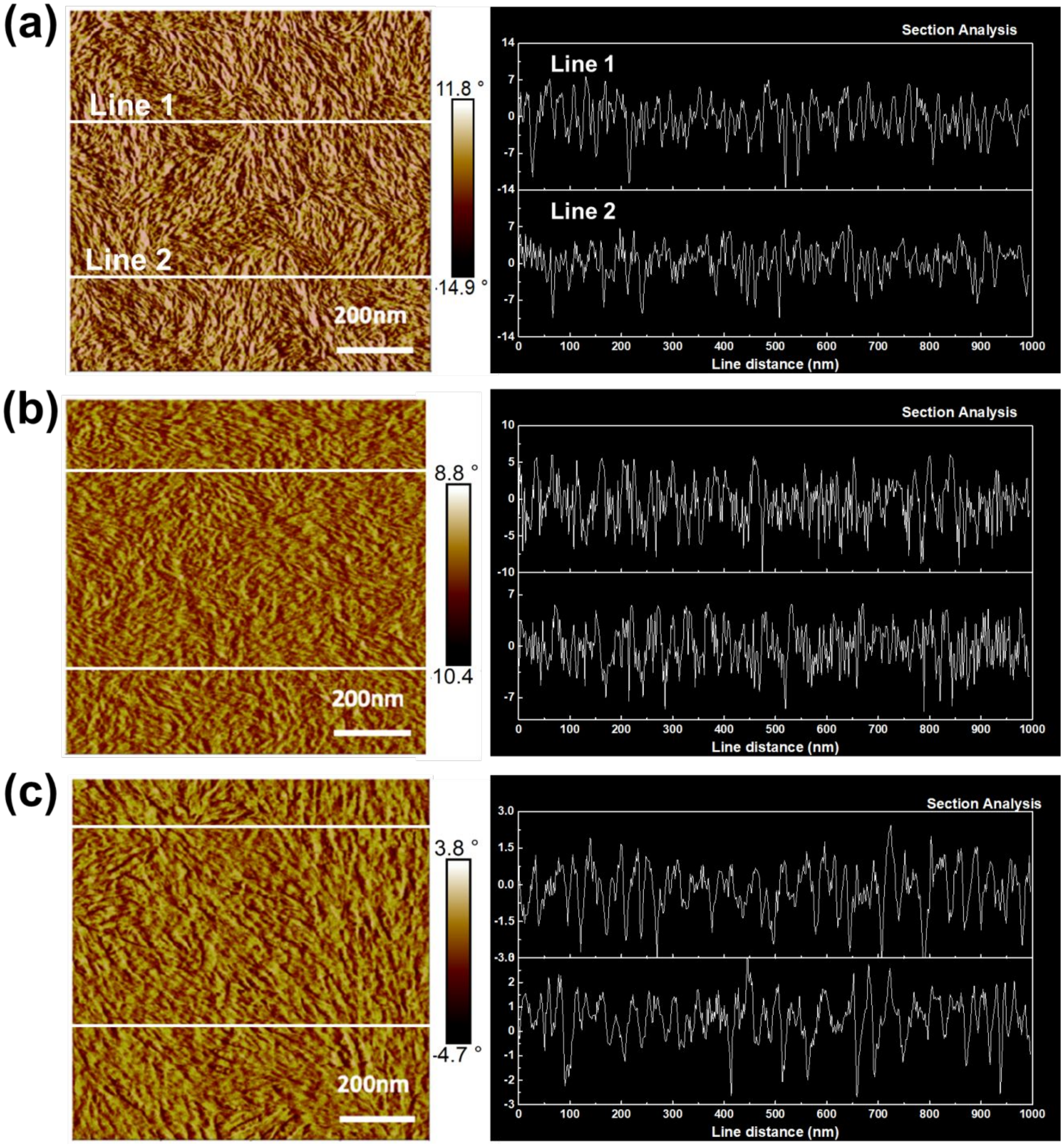

Figure S6. AFM 2D phases images and their corresponding line profiles obtained from section analysis for P(NDI2OD-T2) films: (a) as-cast film from a solution with MW heating, (b) as-cast film from a solution without MW heating, and (c) post-annealed film from a solution without MW heating at 170 °C for 1 h. Each line profile was extracted from the white direction line in the AFM height images. The fibril diameters were estimated by FWHM of each peak in the line profiles.

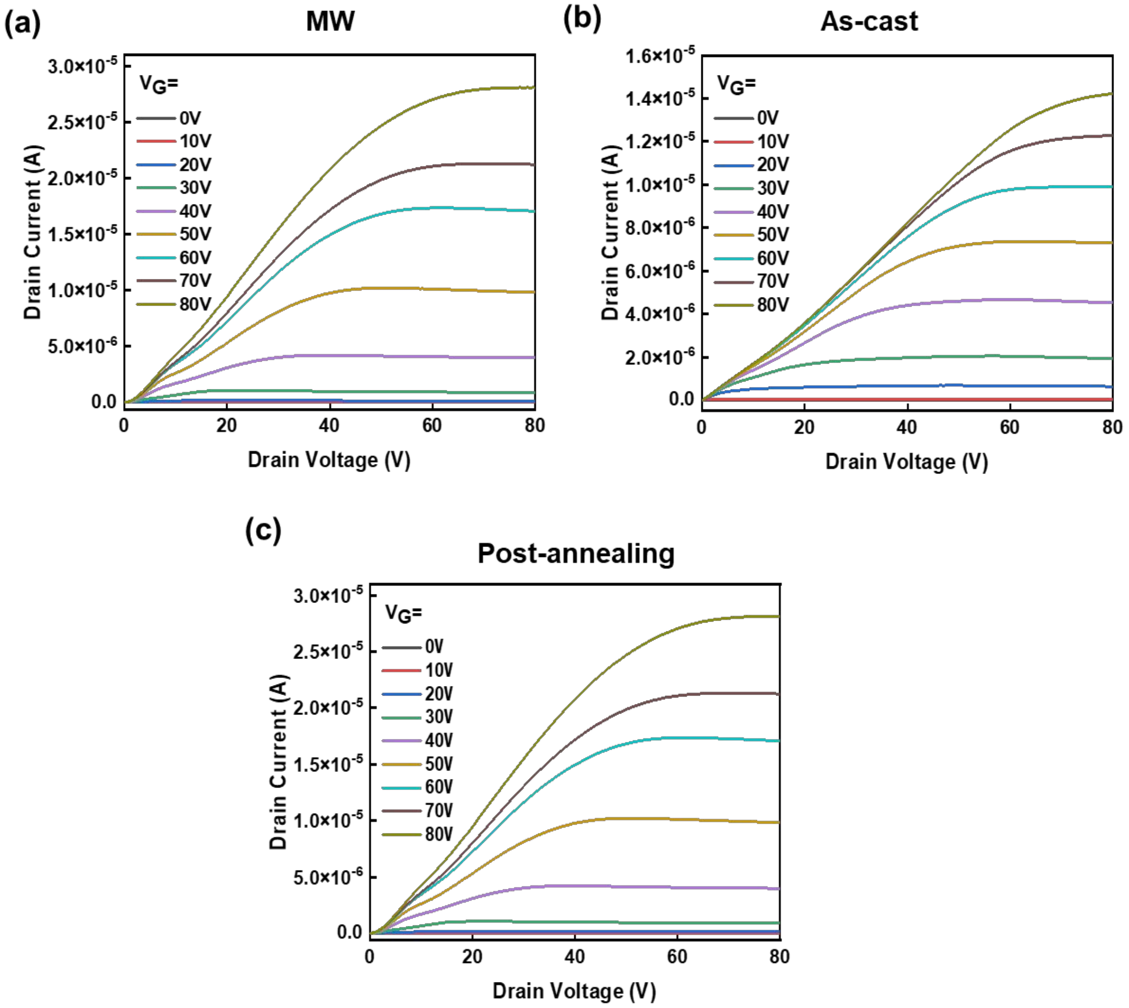

Figure S7. Representative output curves of P(NDI2OD-T2) OFETs with different processing active layer films from: (a) a solution with MW heating, (b), as-cast film from a solution without MW heating and (c) post-annealing of film from a solution without MW heating at 170 °C for 1 h.

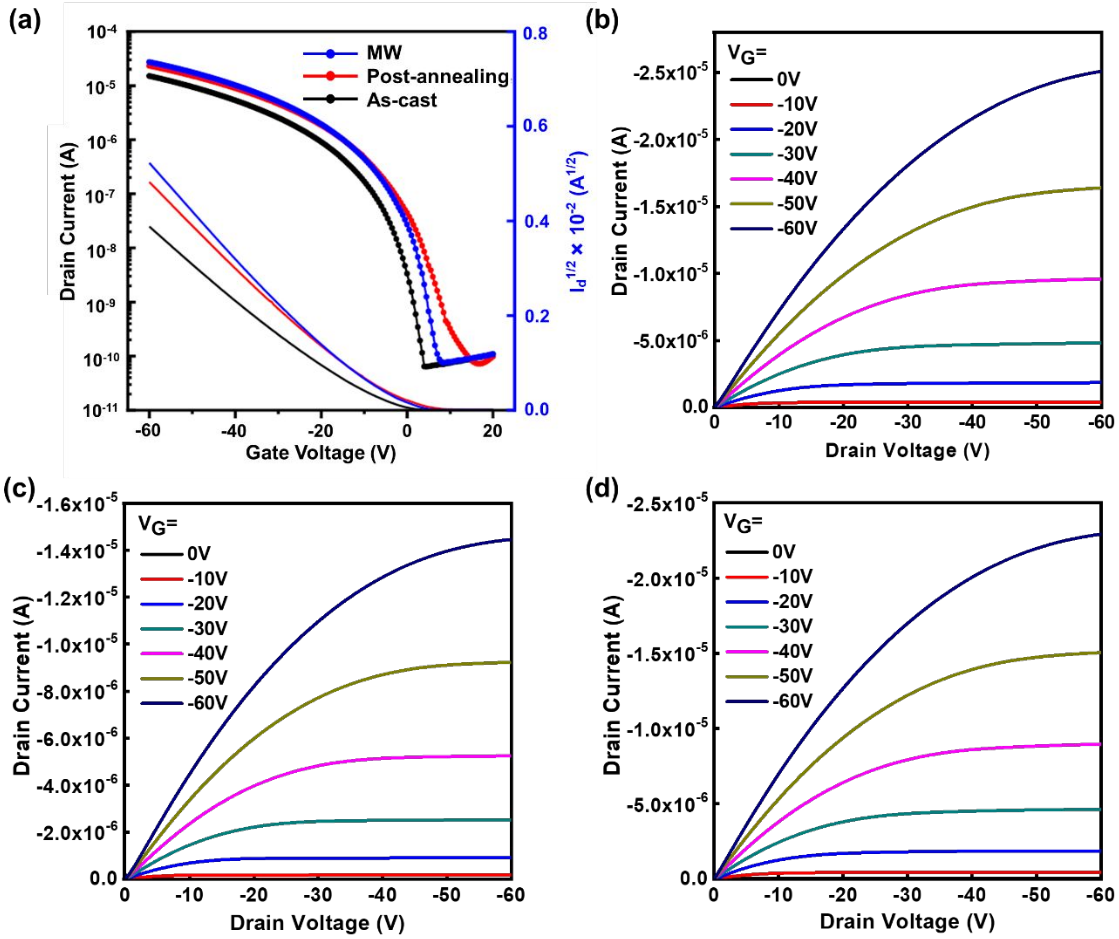

Figure S8. Representative (a) transfer curves and (b-d) output curves of P3HT transistor with different preparation of films from a solution with MW heating, as-cast film from a solution without MW heating or post-annealing, and post-annealing of film from a solution at 170 °C for 1 h.

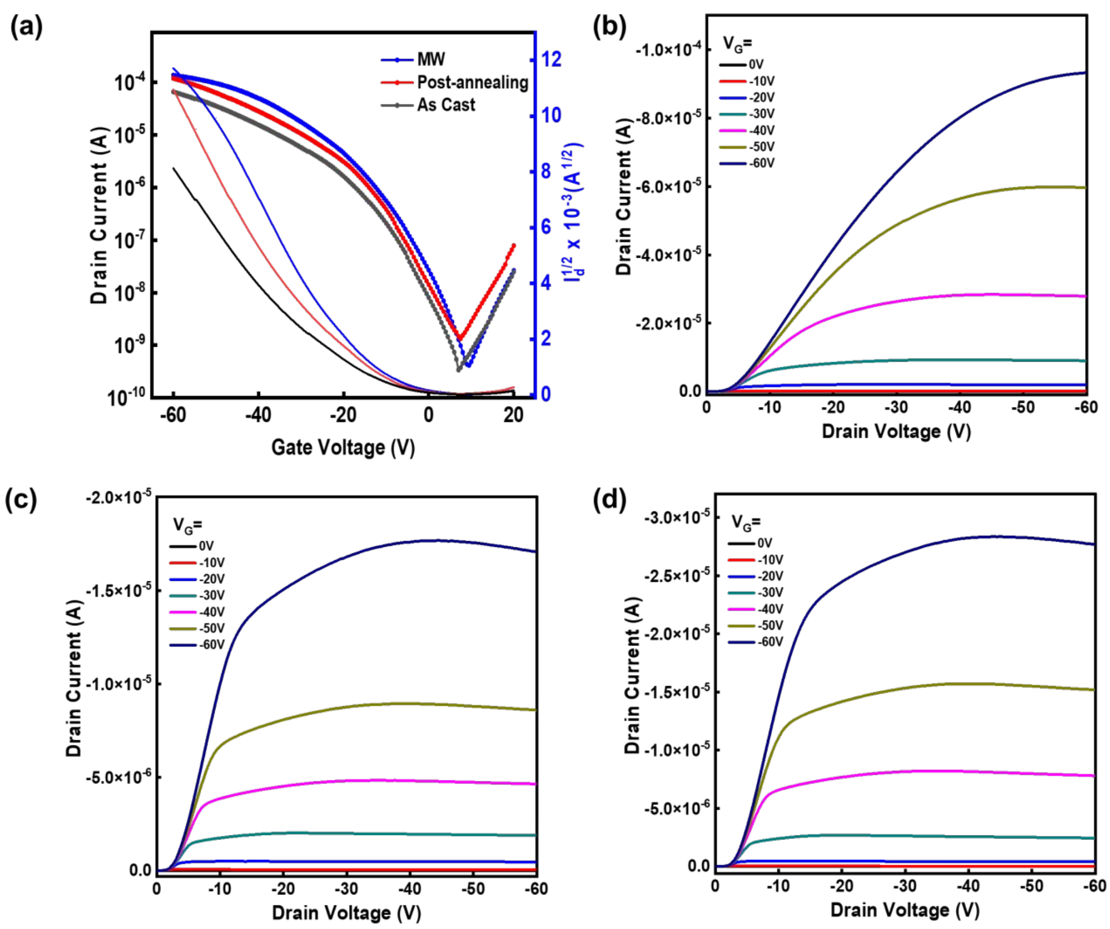

Figure S9. Representative (a) transfer curves and (b-d) output curves of PDPP2T transistor with different preparation of films from a solution with MW heating, as-cast film from a solution without MW heating or post-annealing, and post-annealing of film from a solution at 170 °C for 1 h.

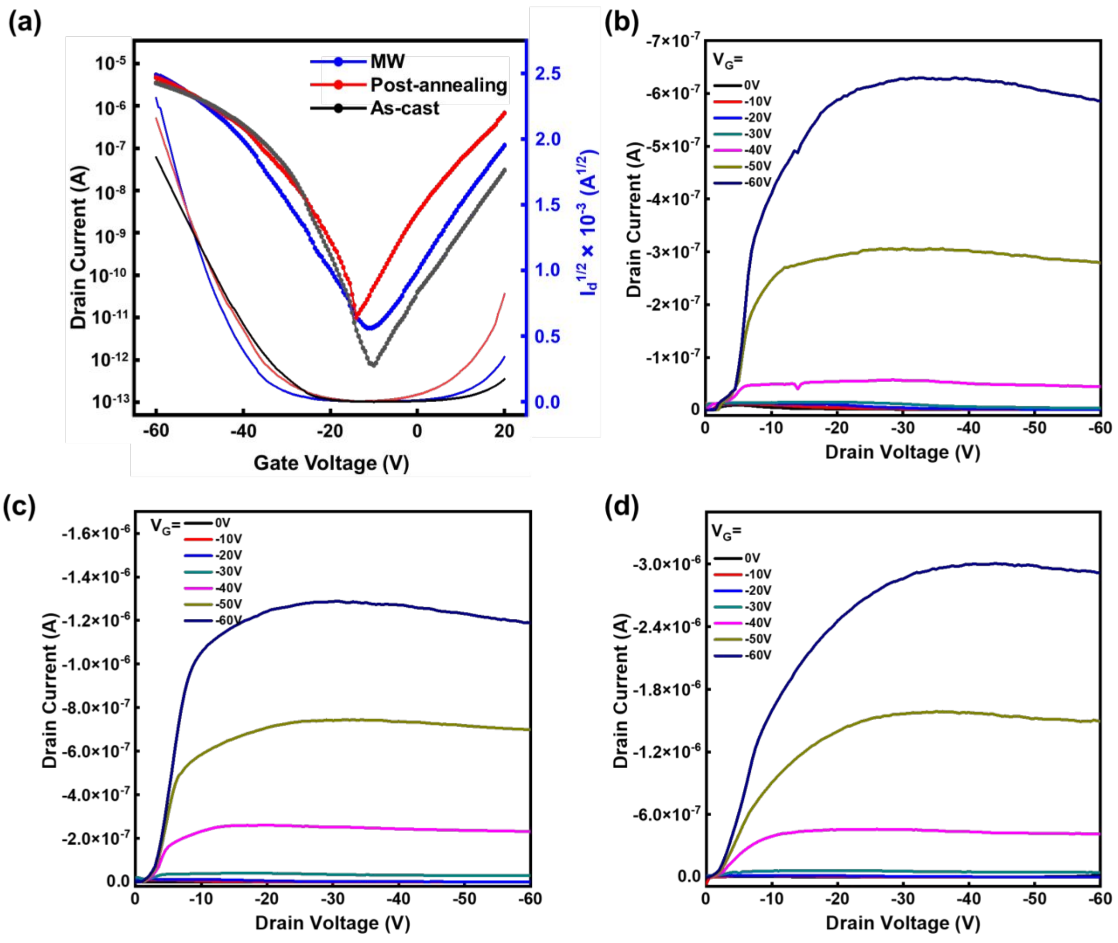

Figure S10. Representative (a) transfer curves and (b-d) output curves of P(IID-BT) transistor with different preparation of films from a solution with MW heating, as-cast film from a solution without MW heating or post-annealing, and post-annealing of film from a solution at 170 °C for 1 h.

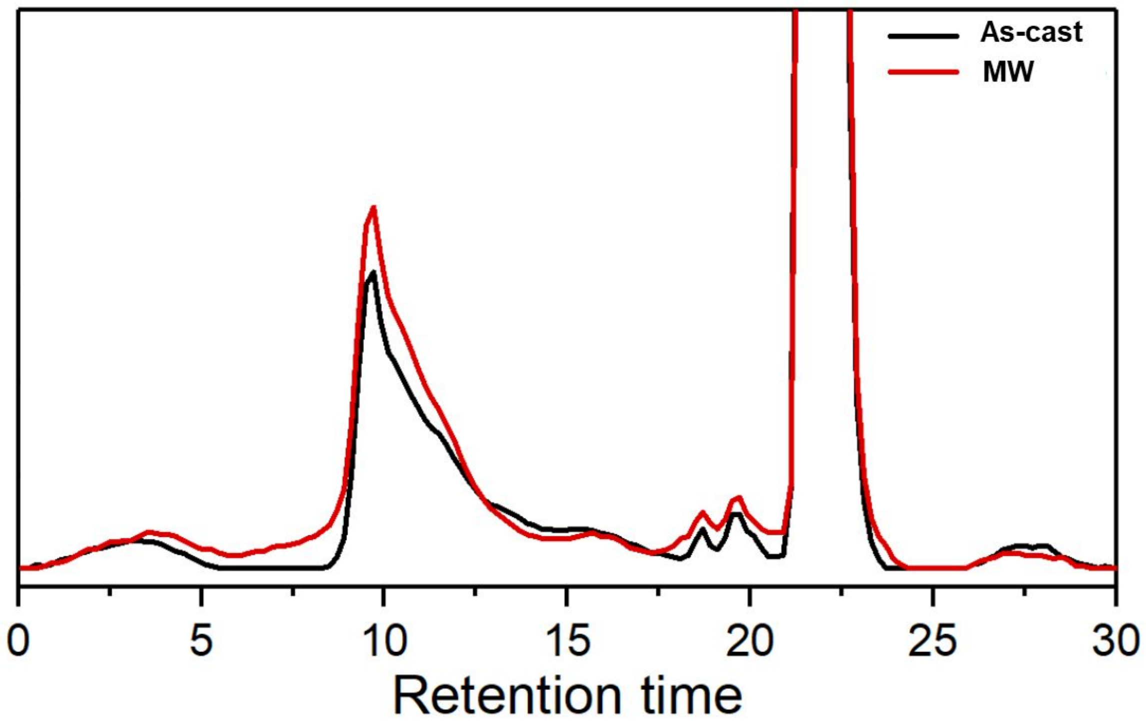

Figure S11. Gel permeation chromatography (GPC) traces of PNDI2OD-T2 in chlorobenzene.

Table S1. The FWHM and crystalline coherence length of P(NDI2OD-T2) based on GIWAXS parameters.

Process condition	Lamellar spacing (*h00*) [Å]	FWHM	Average crystallite size (nm)
**MW**	25.12	0.4418	13.2
**As-cast**	24.47	0.4123	14.2
**Post-annealing**	25.12	0.5006	11.7

The crystalline coherence length was calculated based on Scherrer's equation:

$$D = K\lambda/\beta\cos(\theta)$$

where D is average crystallite size and K is Scherrer constant ≈0.93, λ is the wavelength of the X-ray beam, β is the Full width at half maximum (FWHM) of the peak, and θ is the Bragg angle.

Table S2. The electrical characteristics of P3HT, DPP2T and IID-BT OFETs.

Conjugated Polymer	Process condition	Average field-effect mobility ($cm^2 V^{-1} s^{-1}$)	V_{th} (V)	ON/OFF ratio
**P3HT**	MW	1.01×10^{-1}	-8.73	5.5×10^5
	Post-annealing	8.72×10^{-2}	-7.36	5.5×10^5
	As-cast	6.42×10^{-2}	-11.70	6.9×10^5
**PDPP2T**	MW	8.98×10^{-1}	-17.39	3.9×10^5
	Post-annealing	8.79×10^{-1}	-27.18	1.8×10^5
	As-cast	5.09×10^{-1}	-24.87	2.8×10^5
**P(IID-BT)**	MW	1.36×10^{-1}	-41.22	8.2×10^5
	Post-annealing	9.36×10^{-2}	-34.83	5.5×10^5
	As-cast	4.80×10^{-2}	-31.64	4.0×10^6
